# The association of *GATM* polymorphism with statin-induced myopathy: a systematic review and meta-analysis

**DOI:** 10.1007/s00228-020-03019-3

**Published:** 2020-10-13

**Authors:** Mengyuan Liu, Fangfang Fan, Yan Zhang, Jianping Li

**Affiliations:** Department of Cardiology, Peking University First Hospital, No. 8 Xishiku St, Xicheng District, Beijing, 100034 China

**Keywords:** Glycine amidinotransferase gene, Statin-induced myopathy, Single nucleotide polymorphism, Meta-analysis

## Abstract

**Purpose:**

Statin-induced myopathy (SIM) is the commonest reason for discontinuation of statin therapy. The aim of this present meta-analysis is to assess the relationship between glycine amidinotransferase gene (*GATM*) polymorphism and risk of SIM.

**Methods:**

MEDLINE, EMBASE, Web of Science, and Cochrane Library databases were searched systematically for case-control studies investigating the relationship between *GATM* polymorphism and SIM. Retrieved articles were carefully reviewed and assessed according to the inclusion criteria. Associations were assessed in pooled data by calculating odds ratio with 95% confidence intervals. Subgroup analysis was performed according to comedications and severity of SIM.

**Results:**

Six studies with 707 cases and 2321 controls were included in this meta-analysis. *GATM* rs9806699 G>A was associated with decreased risk of SIM (OR = 0.80, 95% CI 0.68–0.94, *P* = 0.006). This association remained significant in the subgroup with fibrates or niacin excluded. However, the association of rs9806699 G>A with severe SIM was not significant. In addition, another two variations at *GATM*, rs1719247 C>T, and rs1346268 T>C were also associated with declined risk of SIM.

**Conclusions:**

*GATM* polymorphism including rs9806699 G>A, rs1719247 C>T, and rs1346268 T>C may be protective factors of SIM. *GATM* rs9806699 G>A may only exert protective effect on mild SIM cases. Our meta-analysis indicates that *GATM* polymorphism may represent a pharmacogenomics biomarker for predicting incidence of SIM, which contributes to risk stratification and optimizing statin adherence.

**Electronic supplementary material:**

The online version of this article (10.1007/s00228-020-03019-3) contains supplementary material, which is available to authorized users.

## Introduction

Statin-induced myopathy (SIM) is the most frequently reported adverse effect of statins and is the commonest cause for discontinuation of statin therapy [1]. Symptoms of SIM can vary from mild myalgia to rare but life-threatening rhabdomyolysis [2]. The high prevalence of statin use made the absolute number of SIM became more substantial. Unfortunately, the underlying mechanisms of SIM have not been fully understood. Risk factors of SIM have been investigated in the past, such as high statin doses, older age, hypothyroidism, hepatic, and renal insufficiency [3–5]. Recently, genetic predisposition was found to play a crucial role in SIM [6]. It is now well established that a single nucleotide polymorphism (SNP) of solute carrier organic anion transporter family member 1B1 (*SLCO1B1*), rs4149056 T > C, increases the risk of SIM [7]. The association of SNPs in other genes, including glycine amidinotransferase gene (*GATM*) and risk of SIM, has also aroused researcher’s interest.

*GATM* is located on chromosome 15q15.3 and it encodes a mitochondrial enzyme, L-arginine, glycine amidinotransferase (AGAT), which is a rate-limiting enzyme involved in the biosynthesis of creatine [8]. Creatine is transported to muscle tissues after synthesis in the liver and kidneys, then it is further transformed to creatine phosphate, which is participated in rapid re-synthesis of ATP [9]. It plays a pivotal role in the myocellular energy metabolism. In 2003, Mangravite et al. firstly reported the protective effect of the *GATM* rs9806699 G > A polymorphism on SIM in a case-control study with SIM cases of 72 [10]. The locus rs9806699 is an expression quantitative trait locus (eQTLs) for the *GATM*. The A allele was associated with a decline of *GATM* expression, leading to decline of creatine synthesis. The reduced creatine availability might affect energy metabolism of skeletal muscle cells and thus participate in the pathogenesis of SIM [9]. However, the following researches regarding the effect of rs9806699 G > A on SIM yielded conflict results [11–15]. Furthermore, other two SNPs, rs1719247 and rs1346268, in *GATM* were found to be in linkage disequilibrium with rs9806699 [7, 10], and the effect of these two SNPs have also been investigated though the results are controversial. Therefore, the role of *GATM* polymorphism in SIM remains a much-debated topic to date. The aim of the present study is to perform a meta-analysis to explore the relationship between *GATM* polymorphism and SIM, which help to identify high-risk population for SIM and provide more individualized recommendation for statin users.

## Methods

This meta-analysis was reported in accordance with the Meta-analysis Of Observational Studies in Epidemiology (MOOSE) guidelines [16]. The MOOSE checklist could be found in the Supplementary Materials A (eTable 1).

### Literature search

We searched the MEDLINE, EMBASE, Web of Science, and Cochrane Library databases through May 2020 for case-control studies investigating the relationship between *GATM* polymorphism and SIM, with the following search terms: statin, gene encoding glycine amidinotransferase, *GATM*, polymorphism, muscle symptom, myopathy, rhabdomyolysis, and myositis. If available, Medical Subject Headings (MeSH) terms were used together with the free text terms and synonyms. In addition, the reference lists of the eligible studies were screened for additional relevant studies.

### Study selection

We included studies that met the following criteria: (1) case-control studies investigating statin-induced myopathy, (2) genotyping and reporting *GATM* polymorphism in each group, (3) sample size more than 10 patients, (4) published in English, (5) allele frequencies available from the study population. Two reviewers (M.L and F.F) independently screened the titles and abstracts of all retrieved citations to identify studies that potentially met the inclusion criteria. Thereafter, the full texts of potentially relevant studies would be obtained and independently scrutinized by two reviewers (M.L and F.F) based on the above-mentioned inclusion criteria to be finally included. Disagreements were resolved by discussing until a consensus was reached.

### Data extraction

Relevant data of included studies was extracted from eligible studies using a standardized form (Supplementary Material B) by two reviewers (M.L and Y.Z) independently. The following information was extracted from each study: the first author’s name, country, sample size, publication year, type of statins used, presence of interacting medications, diagnosis criteria for SIM, *GATM* polymorphism data (including minor allele frequency (MAF) at different sites), validity of the genotyping method, and Hardy–Weinberg equilibrium (HWE) condition of each group. Disagreements were resolved by discussing until a consensus was reached.

### Study outcome

The occurrence of SIM was studied as outcome. SIM was defined as muscle symptoms or creatine kinase levels > 3 × upper limits of normal (ULN). The diagnosis of severe SIM is according to the specific diagnosis of each included study if it is available.

### Quality assessment

The Newcastle–Ottawa Scale was used to assess the quality of studies and the risk of bias [17]. Studies would be assessed with prespecified criteria in three basic domains, i.e., four stars for selection, two stars for comparability, and three stars for exposure. A score > 7 suggested a low risk of bias, a score between 5 and 7 suggested a moderate risk of bias, and a score < 5 suggested a high risk of bias. Two reviewers (M.L and Y.Z) independently performed the assessment. Disagreements regarding quality assessment were resolved by discussion.

### Statistical analysis

The connection between *GATM* polymorphism and SIM risk was reported as odds ratio (OR) with 95% confidence interval (95% CI), based on allele contrast model. Data was pooled using either random effects model or fixed effects model according to the statistical heterogeneity between studies, which was measured by Cochran *Q* test and the *I*^2^ statistic. Heterogeneity between studies was defined a priori as significant when *I*^2^ statistic ≥ 50% or *P* value of *Q* test < 0.05 [18, 19]. If the effects seemed to be homogeneous, the Mantel–Haenszel fixed-effect model was utilized. Otherwise, the random-effect model was used. The risk of publication bias was evaluated by funnel plot and the asymmetry of the plot distribution indicated the presence of publication bias [20]. Planned subgroup analysis was conducted in regard to the presence or absence of interacting medications, different populations, and diagnosis criteria for SIM according to whether CK elevation was obligatory or not. We also pooled OR for the risk of severe SIM if the data was available. Sensitivity analysis was conducted by excluding individual studies that were considered possible confounders to the result. All statistical analysis was performed with STATA 14.1 software (Stata Corporation, College Station, TX, USA). It is considered to be significant if the two-tailed *P* value was less than 0.05. The graphic compositions were performed by R version 3.3.1 (R Core Team).

## Results

### Study characteristics

**Fig. 1:**
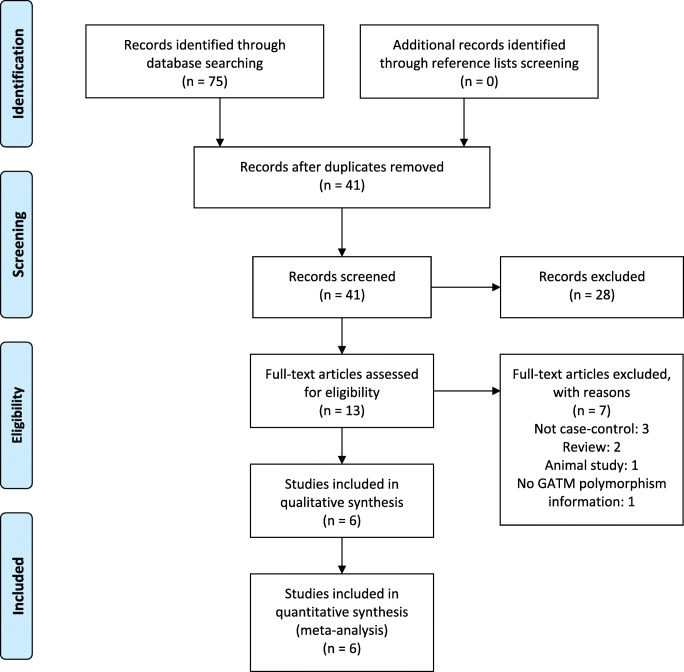
PRISMA flow diagram of the study selection

The PRISMA flow diagram of the study selection was shown in Fig. 1. We retrieved a total of 75 citations from the initial search. After removing the duplicates and screening all titles and abstracts of retrieved citations, 13 articles were further assessed by full-text review according to the above-mentioned inclusion criteria. As a result, 7 full-text articles were excluded due to the reasons shown in the Fig. 1, leaving 6 eligible studies (707 cases and 2321 controls) for final analysis. The characteristics of the included studies were summarized in Table 1. There were two independent populations in the study of Mangravite et al. [10], in which population of Marshfield was genotyped for *GATM* rs9806699, and population of SEARCH trial was genotyped for *GATM* rs1719247 and rs1346268. All of the six included studies had allelic information at rs9806699, while allelic information at rs1719247 and rs1346268 were available in the study of Floyd et al. and two independent population of Mangravite et al. Various statin treatment protocols were applied, and three of six studies excluded fibrates or niacin comedications [10, 12, 13]. The frequencies of the genotypes in three studies followed the HWE, the others were not accessible (Table 1). Three studies had data of severe SIM, and their own definitions of severe SIM were shown on Supplementary Materials A (eTable 2) [11–13]. The Newcastle–Ottawa Scale was used to assess the quality of included studies and the results were shown in Table 2. All the publications received more than six stars, and three of them reached eight stars, which were indicative of high quality.

**Table 1:** Main characteristics of studies included in the meta-analysis

Author	Year	Country	Statin protocol	Interacting medications	The definition of the case	Sample size (*N*)		Hardy–Weinberg equilibrium	SNP in *GATM*
						Case	Control		
Mangravite et al. [10]	2013	USA	Atorvastatin, Simvastatin, or Pravastatin (Marshfield) Simvastatin (SEARCH)	Exclude fibrates or niacin users	CK levels > 3 × ULN and muscle symptoms	72	220	Yes	rs9806699 (Marshfield)
						100	4021 4029	Yes Yes	rs1719247 (SEARCH) rs1346268 (SEARCH)
Carr et al. [11]	2014	UK	Multiple	No	CK levels > 4 × ULN or rhabdomyolysis	150	587	NA	rs9806699
Floyd et al. [12]	2014	USA	Cerivastatin	Exclude fibrates or niacin users	CK levels > 10 × ULN and muscle symptoms	76	643	NA	rs9806699 rs1719247 rs1346268
Luzum et al. [13]	2015	USA	Multiple	Exclude potentially confounding comedications	Muscle symptoms	306	80	Yes	rs9806699
Sai et al. [14]	2016	Japan	Multiple	No	Muscle symptoms	52	86	NA	rs9806699
Bai et al. [15]	2018	China	Rosuvastatin	No	CK > 4 × ULN and/or muscle symptoms	51	705	Yes	rs9806699

*USA*, United States of America; *UK*, United Kingdom; *CK*, creatine kinase; *ULN*, upper limits of normal; *SNP*, single nucleotide polymorphism; *GATM*, glycine amidinotransferase gene

**Table 2:** Quality assessment of included studies by Newcastle–Ottawa Scale

Study	Selection (****)	Comparability (**)	Exposure (***)
Mangravite et al. [10]	***	**	***
Carr et al. [11]	***	*	***
Floyd et al. [12]	***	*	***
Luzum et al. [13]	***	**	***
Sai et al. [14]	***	-	***
Bai et al. [15]	***	**	***

### Meta-analysis results

#### The association between *GATM* rs9806699 and statin-induced myopathy with subgroup analysis

**Fig. 2:**
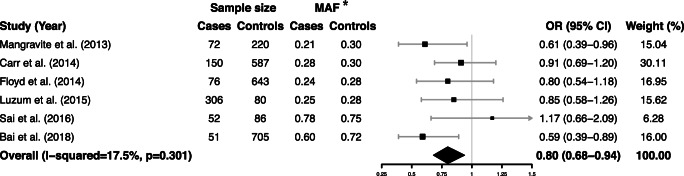
Forest plot of pooled fix-effects-based OR with 95% CI from six studies of association between *GATM* rs9806699 G>A and SIM, comparing SIM case versus control. *MAF at rs9806699 refers to allele frequency for A allele. CI, confidence interval; OR, odds ratio; SIM, statin-induced myopathy; *GATM*, glycine amidinotransferase gene; MAF, minor allele frequency

**Fig. 3:**
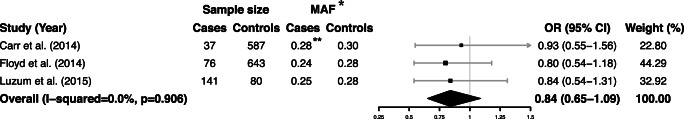
Forest plot of pooled fix-effects-based OR with 95% CI from three studies of association between *GATM* rs9806699 G>A and severe SIM, assessing severe SIM subgroup versus control. *MAF at rs9806699 refers to allele frequency for A allele. **This value was calculated by odds ratio (OR) of MAF between cases and control (OR = 0.94). CI, confidence interval; OR, odds ratio; SIM, statin-induced myopathy; *GATM*, glycine amidinotransferase gene; MAF, minor allele frequency

**Fig. 4:**
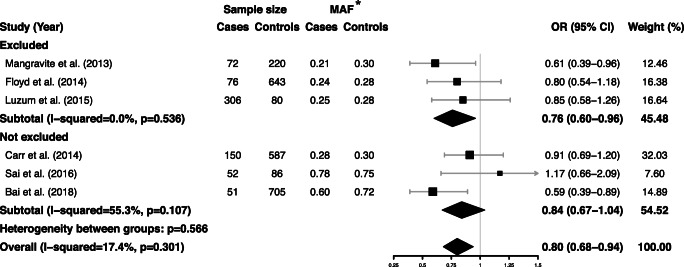
Forest plot of pooled fix-effects-based OR with 95% CI from six studies of association between *GATM* rs9806699 G>A and SIM in subgroup with fibrates or niacin comedications excluded or not, assessing case versus control. *MAF at rs9806699 refers to allele frequency for A allele. CI, confidence interval; OR, odds ratio; SIM, statin-induced myopathy; *GATM*, glycine amidinotransferase gene; MAF, minor allele frequency

The combined data from six eligible case-control studies proved that *GATM* rs9806699 G>A had a protective effect against SIM (OR = 0.80, 95% CI 0.68–0.94, *P* = 0.006, *I*^*2*^ = 17.5%). There was no statistically heterogeneity according to the *Q* test (*P* = 0.301) and the *I*^*2*^ statistic (Fig. 2). The effect of *GATM* rs9806699 G>A was further evaluated in a subgroup analysis of severe SIM. When the three eligible studies with severe SIM subgroups were pooled, the *GATM* rs9806699 polymorphism was found to be not associated with a risk of severe SIM (OR = 0.84, 95% CI 0.65–1.09, *P* = 0.187, *I*^*2*^ = 0%) (Fig. 3). Then, in order to exclude the influence of fibrates or niacin, we assessed the effect of *GATM* rs9806699 G>A in subgroups with fibrates or niacin comedications excluded or not (Fig. 4). The results reached statistically significant in the subgroup with fibrates or niacin comedications excluded (OR = 0.76, 95% CI 0.60–0.96, *P* = 0.023, *I*^*2*^ = 0%), but not significant in the subgroup that not excluding fibrates or niacin uses (OR = 0.84, 95% CI 0.67–1.04, *P* = 0.102, *I*^*2*^ = 55.3%).

The effect of *GATM* rs9806699 G>A was also analyzed in different populations (eFig. 1). The result was statistically significant in the western subgroup (OR = 0.82, 95% CI 0.68–0.98, *P* = 0.030, *I*^2^ = 0%), but not significant in the Asian subgroup (OR = 0.74, 95% CI 0.53–1.04, *P* = 0.081, I^2^ = 72.7%). *GATM* rs9806699 G>A was found to be associated with decreased risk of SIM only in studies using elevation of CK levels as a necessary condition for diagnosis criteria of SIM (OR = 0.81, 95% CI 0.66–0.99, *P* = 0.042, *I*^*2*^ = 4.3%) (eFig. 2).

#### The association between *GATM* rs1719247 and rs1346268 with statin-induced myopathy

**Fig. 5:**
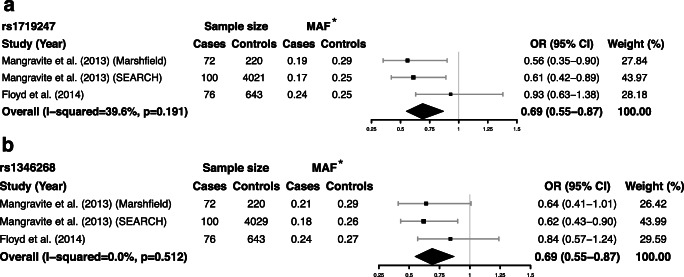
Forest plot of pooled fix-effects-based OR with 95% CI from three studies of association between *GATM* rs1719247 (**a**), *GATM* rs1346268 (**b**), and SIM, comparing SIM case versus control. *MAF at rs1719247 and rs1346268 refers to allele frequency for T and C allele, respectively. CI, confidence interval; OR, odds ratio; SIM, statin-induced myopathy; *GATM*, glycine amidinotransferase gene; MAF, minor allele frequency

We further examined variation at the association of other two SNPs, rs1719247 and rs1346268, with risk of SIM. Three studies investigating the association between *GATM* rs1719247 C>T and SIM was included and combined in a fixed-effects meta-analysis (Fig. 5a). The results indicated that variation at rs1719247 C>T was associated with the risk of SIM (OR = 0.69, 95% CI 0.55–0.87, *P* = 0.002, *I*^*2*^ = 39.6%). Similarly, the association of rs1346268 T>C with SIM was also significant (OR = 0.69, 95% CI 0.55–0.87, *P* = 0.002, *I*^*2*^ = 0%) (Fig. 5b). There was no statistical heterogeneity at rs1719247 and rs1346268 (Fig. 5).

#### Sensitivity analysis

Among the six included studies, Floyd’s study evaluated only cerivastatin, which was voluntarily withdrawn due to a high rate of drug-related rhabdomyolysis and no longer available in the clinical practice, while Bai’s study focused on rosuvastatin, which is a hydrophilic type of statins that has been reported to have fewer SIM adverse events than lipophilic statins. Sensitivity analysis was conducted by excluding these two studies, respectively. The effect of *GATM* rs9806699 remained significant after excluding Floyd’s study (OR = 0.80, 95% CI 0.67–0.95, *P* = 0.013) (eFig. 3), and it was marginally significant after excluding Bai’s study (OR = 0.84, 95% CI 0.71–1.00, *P* = 0.051) (eFig. 4).

#### Publication bias

**Fig. 6:**
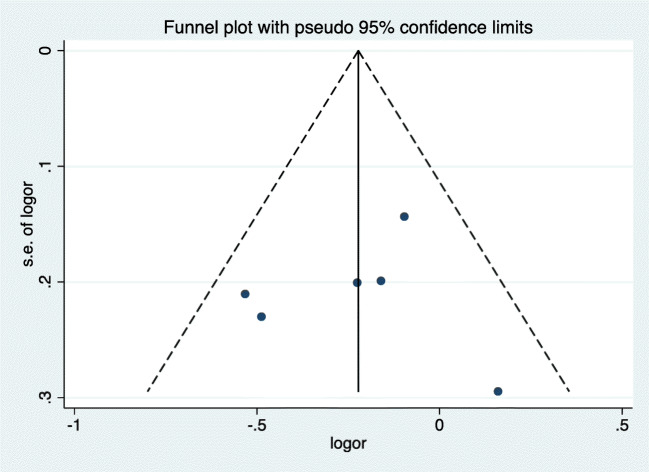
Funnel plots of the meta-analysis of the relationship between *GATM* rs9806699 G>A and SIM. CI, confidence interval; OR, odds ratio; SIM, statin-induced myopathy; *GATM*, glycine amidinotransferase gene

- Publication bias, evaluated by funnel plot, was shown in Fig. 6. All studies were in the 95% confidence limits and the plot showed good symmetry. Therefore, no significant publication bias was detected.

## Discussion

Our meta-analysis showed that *GATM* rs9806699 G>A was associated with decreased risk of SIM. This association remained significant in the subgroup with fibrates or niacin excluded, indicating that *GATM* rs9806699 G>A might be an independent protective factor for SIM. The association of rs9806699 G>A with severe SIM was not significant. Furthermore, other two SNPs of *GATM*, rs1719247 C>T and rs1346268 T>C were also related to reduced risk of SIM. Our meta-analysis addresses the previous controversy and clarified the effect of *GATM* polymorphism on SIM, which may help to better understand the underlying mechanism of SIM and contribute to individual risk stratification for statin users.

A protective effect of *GATM* rs9806699 G>A was observed in our present meta-analysis. This correlation was firstly reported by Mangravite et al. in 2013. By using gene expression profiling of lymphoblastoid cell lines derived from 480 participants treated with simvastatin, they identified that an eQTL for *GATM*, rs9806699, interacted with simvastatin exposure. *GATM* rs9806699 G>A was further found to be associated with decreased incidence of SIM in their study population of 72 cases and 220 controls, which were matched based on statin exposure, age, and gender [10]. However, subsequent studies yielded conflict results [11–15]. Luzum et al. [13] and Sai et al. [14] only used “muscle symptoms” as diagnosis of cases in their study without considering CK levels. Our subgroup analysis indicated that the effects of *GATM* rs9806699 were only significant in studies using CK levels as criteria. Cases in Floyd et al.’s [12] study were all severe cases with CK levels> 10 × ULN and muscle symptoms, while SIM cases of Mangravite et al. were incipient. It was possible that this *GATM* variant only protect against mild but not severe SIM. Other differences in the study populations might also lead to inconsistent results.

The association of variations at another two SNPs, rs1719247 and rs1346268, with SIM has also been investigated. In the Study of Effectiveness of Additional Reductions in Cholesterol and Homocysteine (SEARCH) with 100 myopathy cases, both of variations at rs1719247 and rs1346268 was associated with decreased risk of SIM [10]. Mangravite et al. also verified the protective effect of variation at rs1719247 in another independent population with 72 cases [10]. However, Floyd et al. could not replicate these results in their case-control study [12]. As a result, we included these three studies and verified that of both rs1719247 C>T and rs1346268 T>C were also protective factors for SIM. These two SNPs are in linkage disequilibrium with rs9806699 [10], so the association between these two SNPs and SIM might be similar to that of rs9806699 [21]. But, how variations at these two SNPs affect the expression of GATM and thereby influence the occurrence of SIM is still unclear.

To investigate potential factors modifying the effect of *GATM* polymorphism, we performed subgroup analyses of severe SIM cases, with data from studies of Car et al. [11], Floyd et al. [12], and severe subgroup of Luzum.et al. [13] combined together. No association was found between *GATM* rs9806699 polymorphism and risk of severe SIM with no heterogeneity within included studies. These results indicated that *GATM* rs9806699 G>A might only exert protect effect against mild but not severe SIM. Regarding that all cases in the study of Mangravite et al. [10] were incipient myopathy, it might be explained why their results could not be replicated in case-control studies of severe cases or our subgroup analysis. However, there has been few separate studies for mild cases, and some studies have not distinguished between severe and mild cases, it is difficult for us to perform a subgroup analysis for mild cases with enough studies included. Furthermore, the definition of severe cases was not uniform, which might affect the effect of *GATM* rs9806699 variation. Therefore, a larger study with an accurate definition of the severity of myopathy is required. Because fibrates and niacin were considered to increase incidence of SIM and they were commonly used as comedication of statin in patients with dyslipidemia [10, 22–24], we evaluated the effect of rs9806699 in subgroups excluding fibrates or niacin. We found that the protective effect of rs9806699 G>A was significant without usage of fibrates or niacin. Due to the promoting effect on SIM of fibrates and niacin, they would mask the protective effect of G>A at *GATM* rs9806699. That provided an explanation for our negative pooled results in subgroup that not excluding fibrates or niacin. So, this finding alerted researchers the necessity of excluding the effects of drugs like fibrates or niacin as much as possible in the future. There was a significant association between *GATM* rs9806699 G>A and SIM in western subgroup but not in Asian subgroup. The insufficient studies at present among Asian population might lead to the non-significant result, thus more evidence in Asian population is needed.

Our meta-analysis has some limitations. First, owing to a lack of standard definition of SIM, there is heterogeneity in the diagnosis of SIM in included studies. Larger sample studies with widely accepted definition of SIM are needed to further verify the effect of *GATM* polymorphism. Second, due to the limited studies regarding the association of *GATM* polymorphism and SIM to date, subgroup analysis for potential influencing factors like statin types,or statin doses cannot be performed. Different races with genetic diversity may also be an influencing factor, unfortunately it is not feasible to draw a conclusion because there is not enough data available for subgroup analysis. Lack of original data of individual patient in our meta-analysis restricted further subgroup analyses and interpretation of differences between studies. However, our study is the largest sample size of meta-analysis to investigate the association between *GATM* polymorphisms and SIM, and also the first one to perform subgroup analysis according to the severity of myopathy and comedications. Larger sample studies with standardized genotyping methods at different SNP sites of *GATM* and accurate definition of SIM severity are warranted to further verify its protective effect on SIM. Third, all of the included studies are case-control studies because there is lack of prospective study to date. Therefore, a well-designed prospective study should be performed in the future, with other co-variants like demographic characteristics, statin dose, types, and period of treatment considered.

## Conclusion

In conclusion, our meta-analysis has indicated that *GATM* polymorphism is associated with the risk of SIM. Variations including rs9806699 G>A, rs1719247 C>T. and rs1346268 T>C may be protective factors of SIM. Subgroup analysis has shown that fibrates or niacin may mask its protective effect and should be excluded in future studies. The association of rs9806699 G>A with severe SIM become non-significant in subgroup analysis, indicating that it may only exert protective effect on mild SIM cases. Our study provides new insight into the genetic determinants of SIM and identification of at-risk population, which may help to reduce the incidence of SIM and optimize statin adherence.

## Electronic supplementary material

ESM 1 (DOCX 1438 kb)

ESM 2 (XLSX 13 kb)
