# Supplementary material for: The association of GATM polymorphism with statin-induced myopathy: a systematic review and meta-analysis
Source: Eur J Clin Pharmacol. 2020 Oct 13;77(3):349–57. doi: 10.1007/s00228-020-03019-3 (PMC7867530; doi:10.1007/s00228-020-03019-3)
Supplement: Supplementary file 1 — (DOCX 1438 kb) [file 228_2020_3019_MOESM1_ESM.docx]

**eTable 1. MOOSE checklist**

**eTable 2. The definition of severe SIM in included studies**

| **Study** | **Severe SIM** |
| --- | --- |
| Carr et al. [11] | CK levels > 10×ULN or clinical record of rhabdomyolysis |
| Floyd et al. [12] | CK levels > 10×ULN and muscle symptoms |
| Luzum et al. [13] | Symptoms of incapacitating muscle pain (with weakness or not)  and rhabdomyolysis |

Abbreviations: SIM, statin-induced myopathy; CK, creatine kinase; ULN, upper limits of normal

**eFig. 1.** Forest plot of pooled fix-effects-based OR with 95% CI from six studies of association between *GATM* rs9806699 G＞A and SIM in subgroups of western and Asian population. Abbreviations: CI, confidence interval; OR, odds ratio; SIM, statin-induced myopathy; *GATM*, glycine amidinotransferase gene.

**eFig. 2.** Forest plot of pooled fix-effects-based OR with 95% CI from six studies of association between *GATM* rs9806699 G＞A and SIM in subgroups of diagnosis of SIM*. Abbreviations: CI, confidence interval; CK, creatine kinase; OR, odds ratio; SIM, statin-induced myopathy; *GATM*, glycine amidinotransferase gene. *Subgroups were divided according to whether elevation of CK level was obligatory or not.

**eFig. 3.** Forest plot of pooled fix-effects-based OR with 95% CI for association between *GATM* rs9806699 G＞A and SIM, excluding Floyd’s study. Abbreviations: CI, confidence interval; OR, odds ratio; SIM, statin-induced myopathy; *GATM*, glycine amidinotransferase gene.

**eFig. 4.** Forest plot of pooled fix-effects-based OR with 95% CI for association between *GATM* rs9806699 G＞A and SIM, excluding Bai’s study. Abbreviations: CI, confidence interval; OR, odds ratio; SIM, statin-induced myopathy; *GATM*, glycine amidinotransferase gene.
